# New World Screwworm Infestation in Wild Mountain Tapirs, Central Andes Mountains, Colombia

**DOI:** 10.3201/eid3109.250339

**Published:** 2025-09

**Authors:** Juan Camilo Cepeda-Duque, Leidy Johana Cano-González, Gerardo Elejalde, Juan Camilo Mantilla, Diego Álvarez-Arellano, Julio Cesar Gómez-Salazar, Victoria Rodríguez, Diego J. Lizcano, Jesús Alfredo Cortés-Vecino, Álvaro A. Faccini-Martínez, Thiago Fernandes Martins, Jacob Owens, Jordan Davis-Powell, Liza Dadone, Carlos Galvis, Budhan S. Pukazhenthi, Juliana Vélez

**Affiliations:** Tiger Cats Conservation Initiative, Dosquebradas, Colombia (J.C. Cepeda-Duque); Organización Ambiental Chinampa, Pereira, Colombia (J.C. Cepeda-Duque, L.J. Cano-González, J.C. Mantilla); International Union for Conservation of Nature and Natural Resources Species Survival Commission, Tapir Specialist Group, Gland, Switzerland (J.C. Cepeda-Duque, V. Rodríguez, D.J. Lizcano, J. Owens, J. Davis-Powell, L. Dadone, C. Galvis, B.S. Pukazhenthi, J. Vélez); Laboratorio de Entomología, Corporación Universitaria de Santa Rosa de Cabal, Santa Rosa de Cabal, Colombia (G. Elejalde, D. Álvarez-Arellano); Corporación Autónoma Regional de Risaralda, Pereira (J.C. Gómez-Salazar); Wildlife Conservation Society, Colombia, Cali, Colombia (D.J. Lizcano); Laboratorio de Parasitología Veterinaria, Facultad de Medicina Veterinaria y de Zootecnia, Universidad Nacional de Colombia, Bogota, Colombia (J.A. Cortés-Vecino); Hospital Militar Central, Bogota (Á.A. Faccini-Martínez); Universidad Militar Nueva Granada, Bogota (Á.A. Faccini-Martínez); Faculdade de Medicina Veterinária e Zootecnia, Universidade de São Paulo, Sao Paulo, Brazil (T.F. Martins); Los Angeles Zoo & Botanical Gardens, Los Angeles, California, USA (J. Owens, J. Davis-Powell); Giraffe Veterinary Services, Colorado Springs, Colorado, USA (L. Dadone); Fundación Zoológica de Cali, Cali (C. Galvis); Smithsonian’s National Zoo and Conservation Biology Institute, Front Royal, Virginia, USA (B.S. Pukazhenthi); Center for Conservation Biology, Stanford University, Stanford, California, USA (J. Vélez); The Natural Capital Project, Stanford University, Stanford (J. Vélez)

**Keywords:** Screwworm, parasites, zoonoses, Cochliomyia hominivorax, mountain tapir, Tapirus pinchaque, endangered species, myiasis, Colombia

## Abstract

We describe New World screwworm (*Cochliomyia hominivorax*) infestation in 2 injured mountain tapirs (*Tapirus pinchaque*) from a protected area in the Central Andes, Colombia. Screwworms were not a known threat to mountain tapirs. Community outreach is needed to raise awareness on effects of this parasite on humans, domestic animals, and wildlife.

The New World screwworm (NWS) (*Cochliomyia hominivorax*) is an obligate parasite that requires a living host for larval development (*1*). NWS is endemic in countries in the Caribbean region and in South America, and cases have spread north to Central America (https://www.aphis.usda.gov/livestock-poultry-disease/cattle/ticks/screwworm). Thus, risk for re-introduction of NWS from South America to NWS-free areas in Central and North America is constant (*2*). Because of its substantial effects on livestock, wildlife, and human health, NWS infection is reportable in Colombia (*1*,*3*). However, reports of this parasite affecting the mountain tapir (*Tapirus pinchaque*), an endangered species on the International Union for Conservation of Nature Red List (https://www.iucnredlist.org), have only been anecdotal. Here, we describe 2 cases of myiasis caused by NWS infestation in mountain tapirs in a protected area of the Central Andes of Colombia.

We collected NWS larvae from 2 adult mountain tapirs, 1 female on October 19, 2024, and 1 male on January 28, 2025, in Ucumari Regional Natural Park (4°42′14″N, 75°32′14″W) at an altitude of 2,097 meters. Both tapirs had deep, 8–10-cm long wounds with exposed muscles in their hindquarters, consistent with myiasis caused by NWS larvae feeding on living tissues (Figure 1). Neither tapir received prior treatment or was subsequently monitored, making determination of the cause or progression of their injuries impossible. To collect larvae, the local environmental authority, Corporación Autónoma Regional de Risaralda, chemically restrained the female tapir, but the male tapir exhibited docile behavior in its interactions with the local community and did not require restraint. 

**Figure 1:**
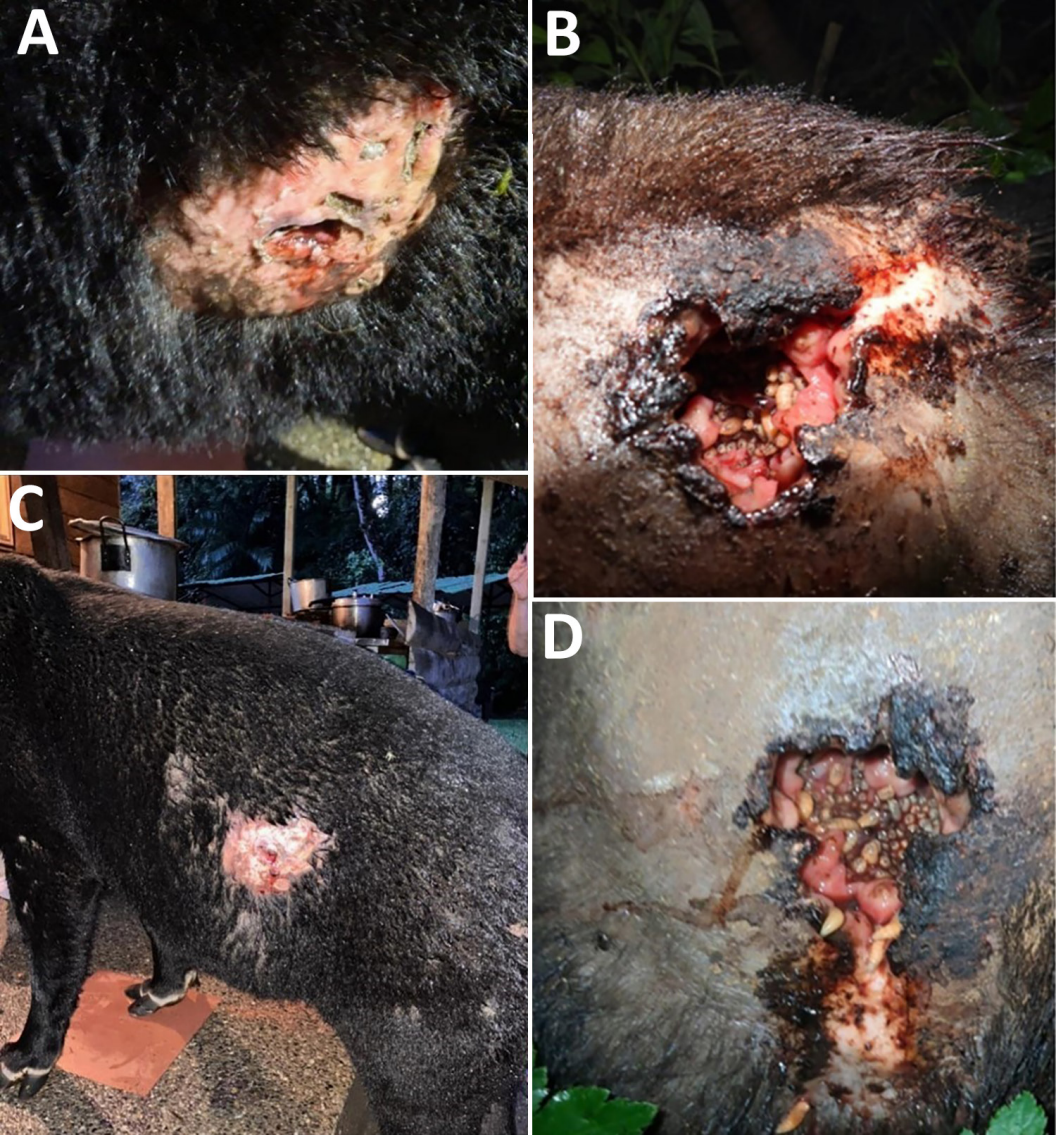
Myiasis in cases of New World screwworm infestation in wild mountain tapirs, Central Andes Mountains, Colombia. Myiasis and active larvae can be seen in large, 8–10-cm wounds on left side of adult male tapir (A, C) and on right hindquarters of adult female tapir (B, D). Both tapirs were in the Ucumari Regional Natural Park, Risaralda, Colombia. We retrieved 2 larvae from the female tapir’s wound and placed in a box until they pupated; after 12 days they emerged as adults (both male) (Appendix Figure 1), which we stored in 96% ethyl alcohol. We collected 20 larvae from the male tapir and stored in 70% ethyl alcohol.

We collected 2 larvae directly from the female tapir’s wound with tweezers and placed larvae in a box until they pupated; after 12 days, they emerged as adult flies (both male) (Appendix Figure 1), which we photographed then stored in 96% ethyl alcohol. We collected 20 larvae from the male tapir and stored larvae in 70% ethyl alcohol. We also photographed phenotypic traits of maggots from the male tapir to enable taxonomic identification and confirm NWS (Figure 2) (*4*). 

**Figure 2:**
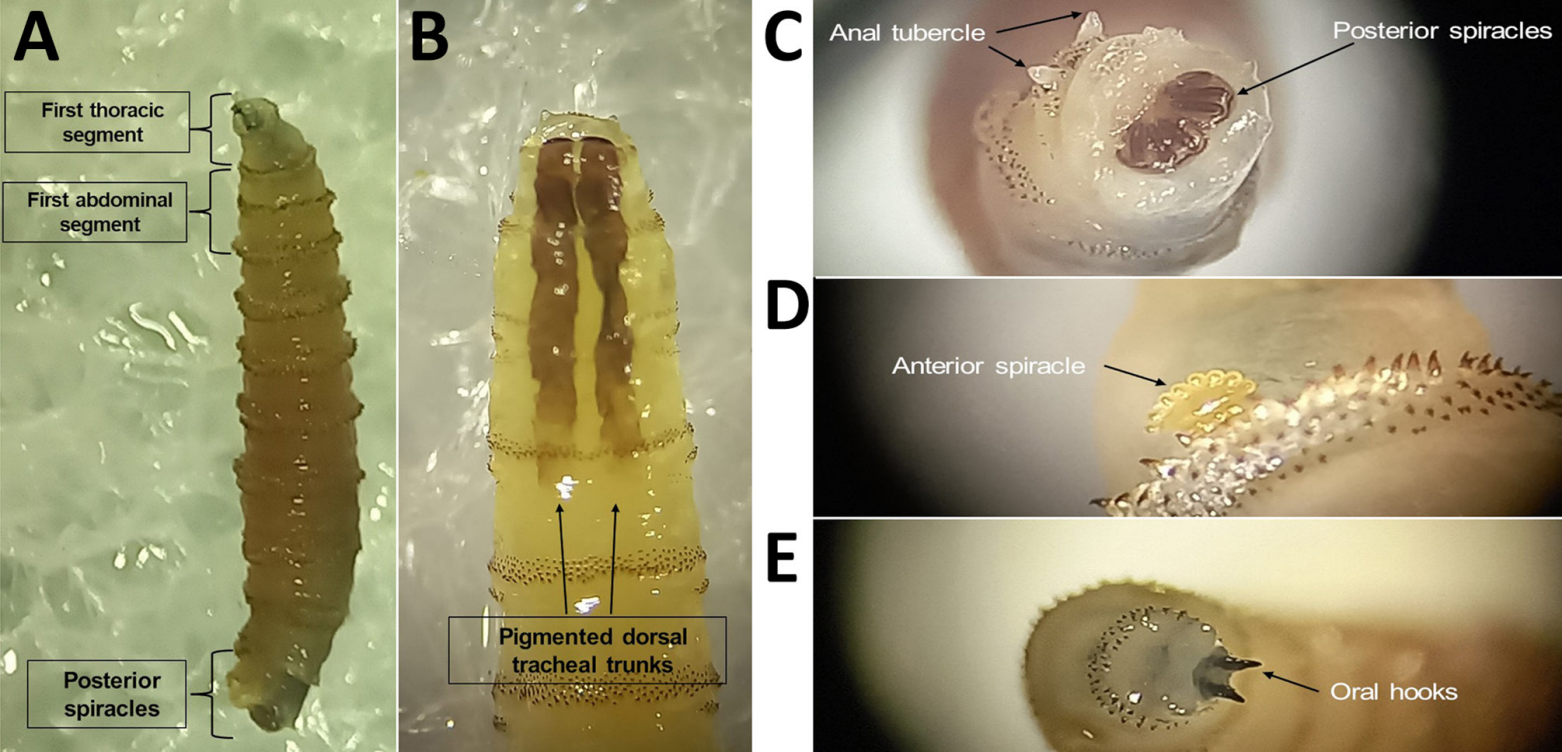
*Cochlyiomia hominivorax* larvae collected from a case of New World screwworm infestation in wild mountain tapir, Central Andes Mountains, Colombia. The larvae were collected from a male tapir and depict features used for taxonomic identification. A) Full larva, showing morphologic features; original magnification ×5. B) First thoracic section, demonstrating pigmented dorsal tracheal trunks; original magnification ×10. C) Posterior spiracles and anal tubercle; original magnification ×25. D) Anterior spiracle; original magnification ×25. E) Oral hooks from first thoracic segment; original magnification ×25. Photographs were taken at the Laboratorio de Parasitología Veterinaria, Universidad Nacional de Colombia, by using an SZX12 stereomicroscope (Olympus, https://www.olympus-lifescience.com).

Our assessment of wild mammals in the area that have potential to host NWS indicated that other threatened species are also at risk, including the clouded tiger-cat (*Leopardus pardinoides*), little red brocket (*Mazama rufina*), northern pudu (*Pudela mephistophiles*), Andean bear (*Tremarctos ornatus*), mountain coati (*Nasuella olivacea*), and Andean squirrel (*Leptosciurus pucheranii*) (Appendix Figure 2). NWS infestations have been documented in several threatened neotropical mammals in the Caribbean and the Americas, including the giant armadillo (*Prionodon maximus*), maned wolf (*Chrysoscyon brachyurus*), jaguar (*Panthera onca*), giant anteater (*Myrmecophaga tridactyla*), lowland tapir (*Tapirus terrestris*), and giant otter (*Pteronura brasiliensis*) (Appendix Table). Humans and domestic animals are also at risk for NWS infestation.

Although often overlooked, myiasis has been linked to severe population declines in wild ungulates, raising conservation concerns for species with low reproductive rates and population sizes, such as mountain tapirs (*5*). For instance, in October 2016, NWS myiasis led to the loss of 14% of the total Key deer (*Odocoileus virginianus clavium*) population in Florida in the United States (*2*). In addition, myiasis resulted in mortality rates of white-tailed deer fawns (*O. v. texanus*) that ranged from 25% to 80% across different regions of the United States (*6*). The proximity of livestock has been associated with NWS outbreaks, and climate change could contribute to expansion of NWS into new areas (*7*).

Factors associated with the emergence of NWS in mountain tapirs remain unclear. Although livestock production in the local area is minimal, contact between livestock and tapirs might exist. Free-ranging dogs, known carriers of NWS (*3*), also have been documented negatively interacting with mountain tapirs (*5*). In addition, intraspecific aggression among tapirs and prolonged use of radio collars also can cause wounds promoting myiasis development, as observed in collared peccaries (*Pecari tajacu*) (*8*) and lowland tapirs (*9*). The parasite also affects humans, and a case of umbilical myiasis was reported in a 7-day-old infant in La Virginia, Risaralda, Colombia, in 2020 (*10*).

To mitigate the threat from NWS, Colombia should consider implementing a biological control program using the sterile insect technique, similar to eradication efforts in North and Central America (*1*). Implementing such a program in Colombia would require studies to assess technical, political (intergovernmental cooperation), economic (cost-sharing), and environmental feasibility. Integrated control measures at smaller scales could help reduce NWS populations to nonthreatening levels (*1*). Threat mitigation strategies should include medical care for infested wildlife and community outreach to raise awareness about the effects of the parasite on humans, domestic animals, and wildlife.

Appendix Additional information on New World screwworm infestation in wild mountain tapirs, Central Andes Mountains, Colombia.
