# Supplementary material for: New World Screwworm Infestation in Wild Mountain Tapirs, Central Andes Mountains, Colombia
Source: Emerg Infect Dis. 2025 Sep;31(9):1871–4. doi: 10.3201/eid3109.250339 (PMC12407199; doi:10.3201/eid3109.250339)
Supplement: Appendix — Additional information on New World screwworm infestation in wild mountain tapirs, Central Andes Mountains, Colombia. [file 25-0339-Techapp-s1.pdf]

Article DOI: https://doi.org/10.3201/eid3109.250339

EID cannot ensure accessibility for supplementary materials supplied by authors. Readers who have difficulty accessing supplementary content should contact the authors for assistance.

New World Screwworm Infestation in Wild Mountain Tapirs, Central Andes, Colombia

Appendix

Appendix Table. Cases of traumatic myiasis induced by the New World screwworm fly (*Cochlyiomia hominivorax*) reported on wild mammals*

Species	IUCN Status	Geographic region	Country	Reference
Odocoileus virginianus	Least Concern	Florida Keys	United States	(*1*)
Didelphis virginiana	Least Concern	South Texas plains	United States	(*2*,*3*)
Didelphis albiventris	Least Concern	Cerrado	Brazil	(*4*)
Didelphis marsupialis	Least Concern	Cerrado	Brazil	(*5*)
Pteronura brasiliensis	Vulnerable	Cerrado	Brazil	(*6*)
Sylvilagus brasiliensis	Least Concern	South Texas plains	United States	(*3*)
Lepus californicus	Least Concern	South Texas plains	United States	(*3*)
Dicotyles tajacu	Least Concern	Pantanal	Brazil	(*7*)
Tapirus terrestris	Vulnerable	Pantanal	Brazil	(*8*)
Pithecia pithecia	Least Concern	Guiana shield	French Guiana	(*9*)
Priodontes maximus	Vulnerable	Pantanal	Brazil	(*10*)
Alouatta palliata	Least Concern	Barro Colorado Island	Panama	(*11*)
Alouatta seniculus	Least Concern	Guiana shield	French Guiana	(*12*)
Myrmecophaga tridactyla	Vulnerable	Atlantic Forest	Brazil	(*13*)
Chrysocyon brachyurus	Near Threatened	Cerrado	Brazil	(*14*)
Galictis cuja	Least Concern	Not specified	Brazil	(*15*)
Leopardus pardalis	Least Concern	Captive	Venezuela	(*16*)
Coendou prehensilis	Least Concern	Amazon	Brazil	(*17*)
Coendou spinosus	Least Concern	Cerrado	Brazil	(*4*)
Bradypus variegatus	Least Concern	Amazon	Brazil	(*18*)
Hydrochoerus hydrochaeris	Least Concern	Captive	Brazil	(*19*)
Panthera onca	Vulnerable	Pantanal	Brazil	(*20*)
Tapirus pinchaque	Endangered	Andes	Colombia	This study

*IUCN, International Union for the Conservation of Nature.

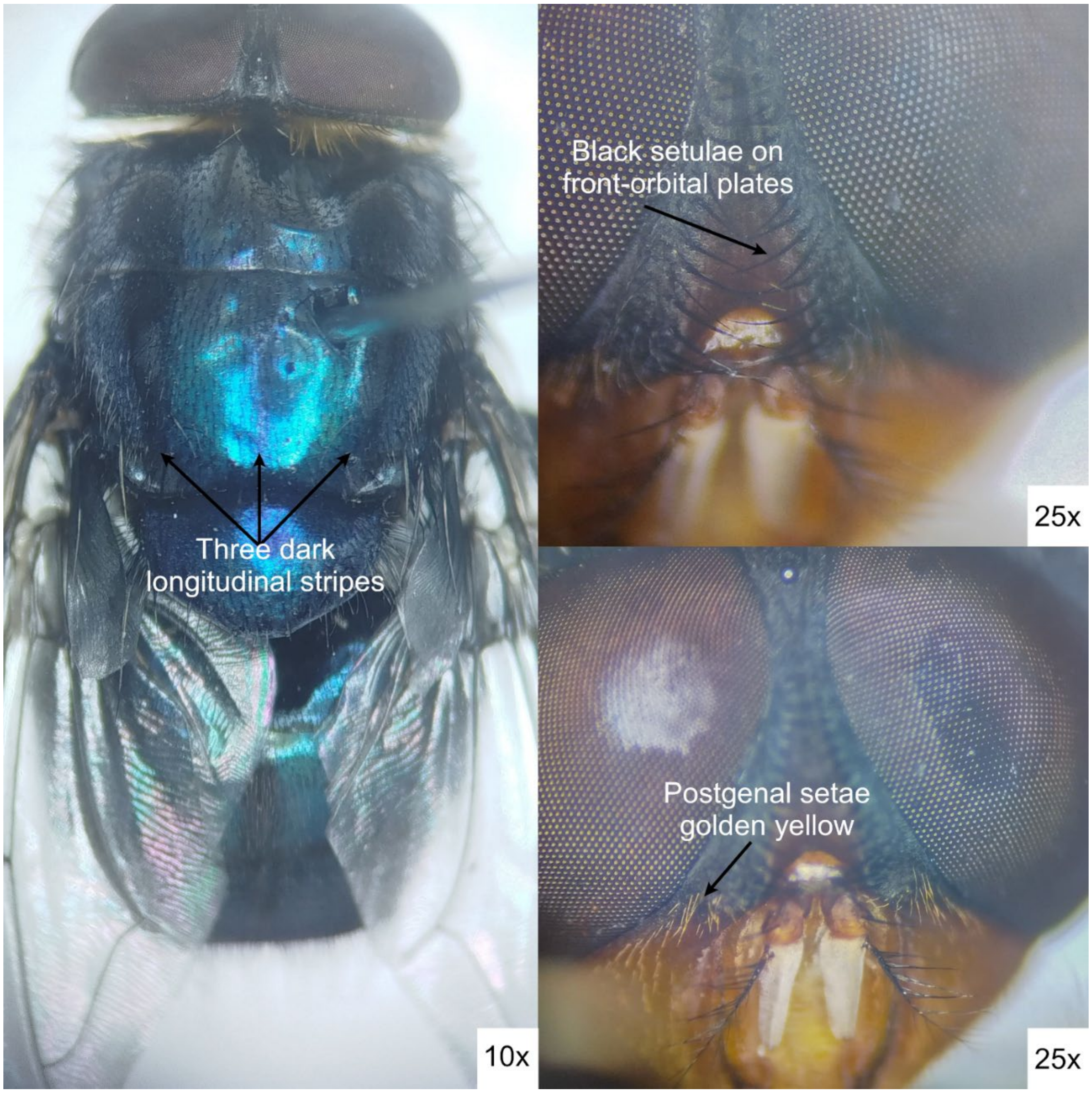

Appendix Figure 1. Images of *Cochlyiomia hominivorax* adult males collected from the female tapir and taken with an Advanced Instruments LTDA JSZ-6s stereomicroscope in which longitudinal stripes on the dorsal surface of the thorax, black setulae on the front-orbital plates of the head, and postgenal setae golden yellow can be seen.

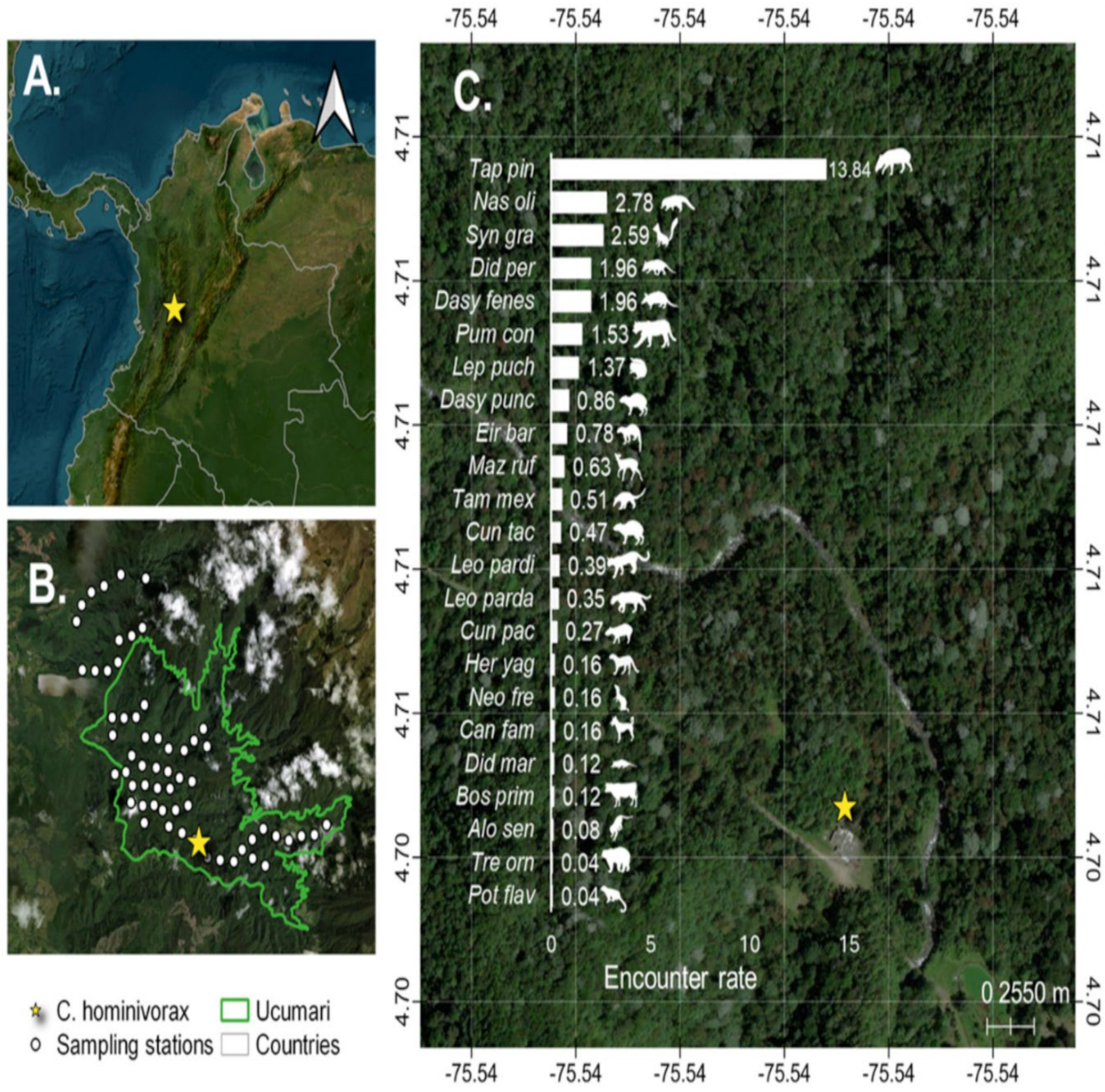

Appendix Figure 2. Camera trap survey in Ucumari Regional Natural Park and the location where the *C. hominivorax* samples were obtained. Encounter rates (C) of native and domestic mammals from a survey with camera trap stations (white symbol) conducted between December 2016–March 2017 in the Ucumari Regional Natural Park. Scientific names of the species are: Tap pin, *Tapirus pinchaque*; Nas oli, *Nasuella olivacea*; Syn gra, *Syntheosciurus granatensis;* Did per, *Didelphis pernigra*; Dasy fenes, *Dasypus fenestratus*; Pum con, *Puma concolor*; Lep puch, *Leptosciurus puchenii*; Dasy punc, *Dasyprocta punctata*; Eir bar, *Eira Barbara*; Maz ruf, *Mazama rufina*; Tam mex, *Tamandua Mexicana*; Cun tac, *Cuniculus taczanowski*; Leo pardi, *Leopardus pardinoides*; Leo parda, *Leopardus pardalis*; Cun pac, *Cuniculus paca*; Her yag, *Herpailurus yagouaroundi*; Neo fre, *Neogale frenata;* Can fam, *Canis familiaris*; Did mar, *Didelphis marsupuialis;* Bos prim, *Bos primigenius*; Alo sen, *Alouatta seniculus*; Tre orn, *Tremarctos ornatus*; Pot flav, *Potos flavus*.

References

1. Skoda SR, Phillips PL, Welch JB. Screwworm (Diptera: Calliphoridae) in the United States: response to and elimination of the 2016–2017 outbreak in Florida. J Med Entomol. 2018;55:777–86. PubMed https://doi.org/10.1093/jme/tjy049

2. Spradbery JP. Screw-worm fly: a tale of two species. Agricultural Zoology Reviews. 1994;6:1–62.

3. Lindquist A. Myiasis of wild animals in southwestern Texas. J Econ Entomol. 1937;30:735–40. https://doi.org/10.1093/jee/30.5.735

4. Ramos RV, Mendes TMF, Hoppe EL, Barros-Battesti DM, Ueta MT, Allegretti SM. Parasite infestations and infections of non-traditional pets and wild mammals: diagnosis and treatment. Rev Bras Parasitol Vet. 2024;33:e017324. PubMed https://doi.org/10.1590/s1984-29612024074

5. Reis FS, Barros MC, Fraga EC, Da Penha TA, Teixeira WC, Dos Santos AC, et al. Ectoparasites of small wild mammals from the adjacent areas of Itapecuru River and Environmental Preservation Area of Inhamum, state of Maranhão, Brazil [in Portuguese]. Rev Bras Parasitol Vet. 2008;17:69–74. PubMed

6. Foerster N, Soresini G, Paiva F, Silva FAD, Leuchtenberger C, Mourão G. First report of myiasis caused by *Cochliomyia hominivorax* in free-ranging giant otter (*Pteronura brasiliensis*). Rev Bras Parasitol Vet. 2022;31:e009522. PubMed https://doi.org/10.1590/s1984-29612022058

7. Medri ÍM, Mourão G. Male-male aggression in free-ranging collared peccaries, *Pecari tajacu* (Artiodactyla, Tayassuidae), from Brazilian Pantanal. Current Ethology. 2016;15:24–9.

8. Quse V, Fernandes-Santos RC. Manual of veterinary medicine for tapers, 2nd edition [in Portuguese]. Gland (Switzerland): International Union for Conservation of Nature Species Survival Commission Tapir Specialist Group; 2014. 165 p.

9. Vié JC, Richard-Hansen C, Fournier-Chambrillon C. Abundance, use of space, and activity patterns of white-faced sakis (*Pithecia pithecia*) in French Guiana. Am J Primatol. 2001;55:203–21. PubMed https://doi.org/10.1002/ajp.1055

10. Kluyber D, Desbiez AL. Medicine of giant armadillos (*Priodontes maximus*). In: Miller RE, Lamberski N, Calle PP, editors. Fowler's zoo and wild animal medicine current therapy, volume 10. St. Louis (MO): Elsevier; 2023. p. 727–736.

11. Hopkins ME, Milton K. Adverse effects of ball-chain radio-collars on female mantled howlers (*Alouatta palliata)* in Panama. Int J Primatol. 2016;37:213–24. https://doi.org/10.1007/s10764-016-9896-y

12. Richard-Hansen C, Vié JC, de Thoisy B. Translocation of red howler monkeys (*Alouatta seniculus*) in French Guiana. Biol Conserv. 2000;93:247–53. https://doi.org/10.1016/S0006-3207(99)00136-6

13. Zupirolli GC, Cruvinel CA, Padula K, Rosa MMC, Zupirolli LAP, Pala EK, et al. Pelvic limb amputation in a giant anteater (*Myrmecophaga tridactyla*) traumatized by an agricultural harvester. Acta Sci Vet. 2021;49:627. https://doi.org/10.22456/1679-9216.141332

14. Cansi ER, Bonorino R, Ataíde HS, Pujol-Luz JR. Myiasis by screw worm Cochliomyia hominivorax (Coquerel) (Diptera: Calliphoridae) in a wild maned wolf *Chrysocyon brachyurus* (Mammalia: Canidae), in Brasília, Brazil. Neotrop Entomol. 2011;40:150–1. PubMed https://doi.org/10.1590/S1519-566X2011000100025

15. Figueiredo MA, Santos ACG, Guerra RDMS. Ectoparasites of wild animals in no Maranhão [in Portuguese]. Pesqui Vet Bras. 2010;30:988–90. https://doi.org/10.1590/S0100-736X2010001100013

16. Pulgar E, Quijada J, Bethencourt A, de Román EM. Report of a case of myiasis caused by *Cochliomyia hominivorax* (Coquerel, 1858) (Diptera: Calliphoridae) in a captive ocelot (*Leopardus pardalis*, Linnaeus, 1758) treated with doramectin [in Spanish]. Entomotrópica. 2009;24:129–33.

17. Lacey LA, George TK. Myiasis in an Amazonian porcupine. Entomol News. 1981;92:79–80.

18. Mastrangelo T, Fresia P, Lyra ML, Rodrigues RA, Azeredo-Espin AML. Genetic diversity and population structure of the New World screwworm fly from the Amazon region of Brazil. Acta Trop. 2014;138(Suppl):S26–33. PubMed https://doi.org/10.1016/j.actatropica.2014.04.002

19. Sanavria A, de Morais MC. Myiasis caused by *Cochliomyia hominivorax* in capybara (*Hydrochoerus hydrochaeris*) in captivity. Rev Bras Med Vet. 1999;21:263–4.

20. May-Junior JA, Fagundes-Moreira R, Souza VBD, Almeida BAD, Haberfeld MB, Sartorelo LR, et al. Dermatobiosis in *Panthera onca*: first description and multinomial logistic regression to estimate and predict parasitism in captured wild animals. Rev Bras Parasitol Vet. 2021;30:e023820. PubMed https://doi.org/10.1590/s1984-29612021003
